# Schwangerschaftsgruppen, neue Gruppe B (Verdacht): MAK-Begründung

**DOI:** 10.34865/mbschwgrbverd11_2or

**Published:** 2026-06-30

**Authors:** Andrea Hartwig, MAK Commission

**Affiliations:** 1 Institut für Angewandte Biowissenschaften, Abteilung Lebensmittelchemie und Toxikologie, Karlsruher Institut für Technologie (KIT) Adenauerring 20a, Geb. 50.41 76131 Karlsruhe Deutschland; 2 Ständige Senatskommission zur Prüfung gesundheitsschädlicher Arbeitsstoffe, Deutsche Forschungsgemeinschaft, Kennedyallee 40, 53175 Bonn, Deutschland. Weitere Informationen: Ständige Senatskommission zur Prüfung gesundheitsschädlicher Arbeitsstoffe | DFG

**Keywords:** Schwangerschaftsgruppe, Gruppe B (Verdacht), Entwicklungstoxizität, Xylidin-Isomere, Vanadium, Isofluran

## Abstract

The Permanent Senate Commission for the Investigation of Health Hazards of Chemical Compounds in the Work Area (MAK Commission) derives limit values for hazardous chemical substances in the air and in biological material (MAK and BAT values) to protect employees at the workplace. However, observance of the MAK and BAT values does not guarantee that the unborn child is protected during pregnancy because these limit values are derived without considering this end point. Therefore, the Commission additionally evaluates all substances to determine whether they may potentially harm a foetus and assigns them to pregnancy risk groups based on the MAK/BAT values. The Pregnancy Risk Groups A, B, and C indicate whether adhering to the MAK or BAT value protects an unborn child. If adequate quantitative data are not available, the substances are assigned to Pregnancy Group D. The Pregnancy Group B (suspected) was introduced for substances that have been found to pose at least a potential hazard of causing developmental toxicity. Data for suspected developmental (neuro)toxic effects are collected for each substance. An expert judgement is prepared to assess the plausibility of a realistic potential hazard for humans at the workplace. Substances for which no MAK or BAT value can be established (such as carcinogenic substances or substances included in Section II b) may likewise be assigned to Pregnancy Group B (suspected).

Die Schwangerschaftsgruppen A, B und C sind Risiko-basierte Gruppen, bei denen ein quantitativer Bezug zwischen dem NOAEL für Entwicklungstoxizität und dem MAK- oder BAT-Wert vorgenommen wird. Ist dieser quantitative Bezug nicht möglich, werden die Stoffe der Schwangerschaftsgruppe D zugeordnet.

Die Schwangerschaftsgruppe D umfasst

- Stoffe, bei denen keine oder keine validen Daten zur Entwicklungstoxizität vorliegen,
- Stoffe mit unvollständiger Untersuchung der Entwicklungstoxizität (z. B. nur Vorliegen einer Screening-Studie nach OECD-Prüfrichtlinie 421 oder 422) und
- Stoffe mit unvollständiger Untersuchung der Entwicklungsneurotoxizität.

Für manche Stoffe, die der Schwangerschaftsgruppe D zugeordnet sind, lässt sich allerdings aufgrund der vorliegenden Daten zumindest ein Gefahrenpotenzial bzw. ein Verdacht auf eine fruchtschädigende Wirkung erkennen, die Daten reichen aber nicht aus, um eine belastbare Risikoabschätzung und damit eine Zuordnung zur Schwangerschaftsgruppe A oder B vorzunehmen.

Ein Verdacht auf eine entwicklungstoxische Wirkung kann sich aus In-vivo-Untersuchungen, meist nach OECD-Prüfrichtlinie 421, 422, 426 oder 443 und evtl. aus In-vitro-Untersuchungen zu mechanistischen Aspekten ergeben, wenn genügend Plausibilität für ein realistisches Gefahrenpotenzial bzw. ein Verdacht auf eine fruchtschädigende Wirkung für den Menschen vorhanden ist. Die Eingruppierung von Stoffen in die neue Gruppe B (Verdacht) soll auf das entwicklungstoxische Gefahrenpotenzial dieser Stoffe hinweisen und ein Signal für einen Abklärungsbedarf setzen.

Diese neue Gruppe B (Verdacht) ist als Verdachtsgruppe neben den bestehenden Risiko-basierten Gruppen A, B und C angesiedelt und zieht eine zusätzliche Signalwirkung nach sich. Die Schwangerschaftsgruppe B entspricht formal der vergebenen Bemerkung „Z“ des Ausschusses für Gefahrstoffe (AGS 2025), was ein Beschäftigungsverbot für Schwangere und Stillende nach sich zieht. Die Schwangerschaftsgruppe B und auch die neue Gruppe B (Verdacht) weisen im Sinne des Mutterschutzgesetzes auf eine unverantwortbare Gefährdung für Schwangere und Stillende hin und dienen neben der Signalwirkung auch der Vorsorge.

Auch Stoffe ohne MAK-Wert (Abschnitt II b oder Kanzerogenitätskategorien 1, 2 bzw. 3), aber mit einem entwicklungs(neuro)toxischen Potenzial, können in die Verdachtsgruppe aufgenommen werden.

## Vorgehensweise bei der Zuordnung zu Gruppe B (Verdacht)

Fehlen quantitative Daten zur Aufstellung eines Risiko-basierten Bezugs zwischen dem NOAEL für Entwicklungs(neuro)toxizität und einem MAK- oder BAT-Wert, kommt der Identifikation des Gefahrenpotenzials für eine fruchtschädigende Wirkung die entscheidende Bedeutung zu. Bei In-vivo-Untersuchungen ist neben der Maternaltoxizität auch die Expositionshöhe, die Applikationsart, die Behandlungsdauer und -häufigkeit sowie ggf. der Abstand zwischen dem LOAEL und dem MAK-Wert zu berücksichtigen. Zudem sind ggf. Daten zum Entstehungsmechanismus sowie zur Humanrelevanz heranzuziehen. Dies kann durch etablierte AOPs (Adverse Outcome Pathway) für entwicklungs(neuro)toxische Wirkungen geschehen. So kann ein Stoff, für den ein MIE (Molecular Initiating Event) aus einem bestehenden AOP nachgewiesen ist, ein Verdachtsstoff sein. Ein Gefahrenpotenzial/Verdacht kann sich auch aus mechanistischen Hinweisen aus In-vivo- oder In-vitro-Untersuchungen ergeben. Zuletzt ist durch Expert-Judgement zu prüfen, ob die Annahme eines realistischen Gefahrenpotenzials bzw. Verdachts für den Menschen ausreichend plausibel ist.

Ein Verdacht auf eine entwicklungsneurotoxische Wirkung trifft im Allgemeinen auf Stoffe zu, für die der kritische Effekt für die MAK- oder BAT-Wert-Ableitung die Neurotoxizität ist oder bei denen sich neonatale bzw. juvenile Tiere im Vergleich zu adulten Tieren als empfindlicher für die substanzinduzierten neurotoxischen Effekte erwiesen haben. Das Gehirn des Menschen weist zum Zeitpunkt der Geburt im Vergleich zum Nager einen höheren Reifegrad auf, der beim Nager erst am 10. Postnataltag erreicht wird (Semple et al. 2013). Folglich sind auch entwicklungsneurotoxische Effekte bei Nagern in der frühen Postnatalzeit als kritisch für die In-utero-Entwicklung beim Menschen anzusehen, und die bei Ratten beobachteten Effekte bis zum 10. Postnataltag zur Bewertung der pränatalen Entwicklungstoxizität beim Menschen zu beachten.

Für eine mögliche Entlassung aus der Gruppe B (Verdacht) kommen belastbare Daten, insbesondere aus In-vivo-Experimenten, zur Speziesübertragung auf den Menschen, zur Toxikokinetik, zum Metabolismus oder Ähnliches in Frage.

### Beispiel für die Zuordnung zu Gruppe B (Verdacht): Xylidin-Isomere (2,3-Xylidin, 2,5-Xylidin, 3,4-Xylidin, 3,5-Xylidin)

Alle vier Xylidin-Isomere führen zur Methämoglobin (MetHb)-Bildung, jedoch in unterschiedlicher Ausprägung (Hartwig und MAK Commission2020 b). Die MetHb-Bildung geht auf die Kooxidation der im Stoffwechsel entstehenden N-Hydroxyderivate und Oxyhämoglobin zurück (Greim 1998). Damit sind die Xylidin-Isomere indirekte MetHb-Bildner.

Eine Erhöhung des MetHb-Spiegels über 1,5 % zeigt beim erwachsenen Menschen eine Exposition gegen MetHb-Bildner an. Gesundheitsschädliche Effekte durch MetHb sind bei gesunden Erwachsenen bis zu einem MetHb-Gehalt von 5 % nicht zu erwarten (Leng und Bolt 2008).

Die Aktivität der NADH-abhängigen MetHb-Reduktase ist in Erythrozyten von Ratten und Mäusen im Vergleich zu denen des Menschen 5- bzw. 10-mal höher. Aufgrund der niedrigeren MetHb-Reduktase-Aktivität beim Menschen sind höhere MetHb-Spiegel die Folge. Beim erwachsenen Menschen ist die MetHb-Reduktase-Aktivität ca. doppelt so hoch wie bei Neugeborenen, was zu höheren MetHb-Spiegeln im Vergleich zu Erwachsenen führt. Bei Ratten und Mäusen hingegen sind die Aktivitäten bei neugeborenen Tieren deutlich höher als bei adulten. Der Mensch verfügt also im Vergleich zu Ratten und Mäusen über eine viel langsamere MetHb-Regeneration, das gilt sowohl für Erwachsene, besonders aber für Neugeborene (Hartwig und MAK Commission2025 e).

Wie die CO-Hb-Bildung (Hartwig 2015) kann die MetHb-Bildung zur Beeinträchtigung der Sauerstoffversorgung des Fetus führen. Unklar ist jedoch, ab welchem MetHb-Gehalt der Mutter es zu einem Sauerstoffmangel des fetalen Gewebes kommen kann.

Alle vier Xylidin-Isomere führen zur MetHb-Bildung, zu hämatologischen Effekten und zu histologischen Veränderungen in Leber (Hämosiderinablagerungen), Milz (Hämosiderinablagerungen), Niere (hyaline Zylinder) und/oder Knochenmark (verstärkte Hämatopoese). Die histologischen Effekte sind größtenteils als Sekundärfolge der Wirkungen auf das Blut anzusehen (Hartwig und MAK Commission2020 b). Damit ist ersichtlich, dass hämatotoxische Effekte und deren Folgeerscheinungen bei allen vier Isomeren im Vordergrund stehen.

Zur pränatalen Entwicklungstoxizität liegen zu keinem der Xylidin-Isomere Daten vor. Aufgrund ihrer genotoxischen Wirkung kann für die Xylidin-Isomere kein MAK-Wert abgeleitet werden. Daher ist die Zuordnung zu einer Risiko-basierten Schwangerschaftsgruppe nicht möglich. Da die NOAEC für den MetHb-Gehalt in Bezug auf die entwicklungstoxische Wirkung beim Menschen nicht bekannt ist, und der menschliche Fetus MetHb deutlich langsamer regenerieren kann als der Erwachsene, besteht die Möglichkeit einer Gefährdung des Ungeborenen durch eine Sauerstoffunterversorgung. Somit lässt sich ein Verdacht auf eine entwicklungstoxische Wirkung begründen, und für die Xylidin-Isomere wird eine Zuordnung zur Schwangerschaftsgruppe B (Verdacht) vorgenommen (Hartwig und MAK Commission2026 e).

## Geprüfte Stoffe seit Einführung der Gruppe B (Verdacht)

Seit 2024 sind neben den Xylidin-Isomeren folgende Stoffe hinsichtlich einer Zuordnung zu Gruppe B (Verdacht) geprüft, aber nicht dieser Gruppe zugeordnet worden:

Allylpropyldisulfid [2179-59-1] (Hartwig und MAK Commission2025 d)

Benzophenon-3 (Oxybenzon) [131-57-7] (Hartwig und MAK Commission2026 b)

Benzylalkoholmono(poly)hemiformal [14548-60-8] (Hartwig und MAK Commission2026 c)

N,N-Bis(2-ethylhexyl)-((1,2,4-triazol-1-yl)methyl)amin [91273-04-0] (Hartwig und MAK Commission2026 d)

1,3-Bis(hydroxymethyl)harnstoff [140-95-4] (Hartwig und MAK Commission2025 b)

Bis(morpholino)methan [5625-90-1] (Hartwig und MAK Commission2025 f)

1,1-Dichlorethen [75-35-4] (Hartwig und MAK Commission2025 a)

1,3-Dimethylol-5,5′-dimethylhydantoin [6440-58-0] (Hartwig und MAK Commission2026 a)

Isofluran [26675-46-7] (Hartwig und MAK Commission2022 a)

Methenamin-3-chlorallylchlorid [4080-31-3] (Hartwig und MAK Commission2025 g)

N-Methylolchloracetamid [2832-19-1] (Hartwig und MAK Commission2025 i)

Naphtha (Erdöl), mit Wasserstoff behandelte schwere [64742-48-9] (Hartwig und MAK Commission2025 h)

4-(2-Nitrobutyl)-morpholin und 4,4′-(2-Ethyl-2-nitro-1,3-propandiyl)bismorpholin [2224-44-4, 1854-23-5] (Hartwig und MAK Commission2025 c)

N,N′,N′′-Triethylhexahydro-1,3,5-triazin [7779-27-3] (Hartwig und MAK Commission2025 j)

Vanadium und seine anorganischen Verbindungen (Hartwig und MAK Commission^2023^)

## Geprüfte Stoffbeispiele aus Gruppe D mit Verdacht auf ein entwicklungsneurotoxisches Potenzial

Beispiele für Stoffe, die der Schwangerschaftsgruppe D zugeordnet sind und für die ein Verdacht auf eine entwicklungsneurotoxische Wirkung gegeben ist, sind in Tabelle 1 aufgeführt.

**Tab. 1:** Beispiele für Stoffe, die der Schwangerschaftsgruppe D zugeordnet sind (Stand Juli 2024) und für die ein Verdacht auf eine entwicklungsneurotoxische Wirkung gegeben ist, aber eine Zuordnung zu Schwangerschaftsgruppe B (Verdacht) nicht vorgenommen wurde, weil sich bei der Evaluierung der entsprechende Verdacht nicht erhärtet hat

**Stoff**	**Grund für Verdacht auf entwicklungsneurotoxische Wirkung**	**Abstand zum MAK-Wert**	**Literatur**
**Kritischer Endpunkt für MAK- oder BAT-Wert-Ableitung: Neurotoxizität**			
Triphenylphosphin [603‑35‑0]	kritischer Effekt für MAK-Wert-Ableitung: Ausfall des Patellarreflexes, Hund, Gavage; keine Daten zur Entwicklungsneurotoxizität	keine Angabe möglich	Hartwig und MAK Commission2022 b
Chlormethan [74‑87‑3]	kritischer Effekt für MAK-Wert-Ableitung: Degeneration der granulären Schicht im Kleinhirn, Maus, Inhalation; keine Daten zur Entwicklungsneurotoxizität	keine Angabe möglich	Hartwig und MAK Commission2021 a
Xylol (alle Isomere) [1330‑20‑7]	kritischer Effekt für MAK-Wert-Ableitung: pränarkotische Wirkung, Mensch, Inhalation; keine belastbaren Daten zur Entwicklungsneurotoxizität	keine Angabe möglich	Hartwig und MAK Commission2021 b
Trikresylphosphat, Summe aller o-Isomere [78‑30‑8]	kritischer Effekt für MAK-Wert-Ableitung: klinisch beobachtete Muskelschwäche, Katze, dermal; Mensch: Neuropathien nach oraler u. dermaler Aufnahme; keine Daten zur Entwicklungsneurotoxizität	keine Angabe möglich	Hartwig und MAK Commission2020 a
1,2,4-Triethylbenzol [877‑44‑1]	kritischer Effekt für MAK-Wert-Ableitung: periphere Neurotoxizität (Axonopathien), Ratte, Maus, Gavage; keine Daten zur Entwicklungsneurotoxizität	keine Angabe möglich	Hartwig und MAK Commission^2018^
Trimethylpentan (alle Isomere) [29222‑48‑8]	kritischer Effekt für MAK-Wert-Ableitung: akute neurotoxische Effekte (Reduktion der visuell evozierten Potenziale), Ratte, Inhalation; keine Daten zur Entwicklungsneurotoxizität	keine Angabe möglich	Hartwig und MAK Commission^2017^
Naphtha (Erdöl), mit Wasserstoff behandelte schwere [64742-48-9]	kritischer Effekt für MAK-Wert-Ableitung: Beeinträchtigungen in neuropsychologischen Leistungstests, Mensch, Inhalation; keine belastbaren Daten zur Entwicklungsneurotoxizität; keine experimentellen Daten, die zeigen, dass juvenile Tiere empfindlicher für Naphtha-induzierte neurotoxische Effekte sind als adulte Tiere	keine Angabe möglich	Hartwig 2009
**Kritischer Endpunkt für MAK- oder BAT-Wert-Ableitung: nicht Neurotoxizität**			
Vanadium und seine anorganischen Verbindungen	kritischer Effekt für MAK-Wert-Ableitung: Lungeneffekte, In-vitro- u. In-vivo-Versuche: höhere Empfindlichkeit von Oligodendrozytenvorläuferzellen im Vgl. zu reifen Oligodendrozyten sowie Störungen der Myelinisierung u. motorische Beeinträchtigungen bei neugeborenen/juvenilen Ratten	i.p. Effektdosis (Ratte) 1,25 mg V/kg KG u. Tag (Worst Case); Abschätzung Luftkonzentration von 2,2 mg V/m^3^ (i.p. u. inhalative Aufnahme je 100 %): 440-facher Abstand zum MAK-Wert von 0,005 mg V/m^3^	Hartwig und MAK Commission^2023^
Isofluran [26675-46-7]	kritischer Effekt für MAK-Wert-Ableitung: Effekte auf Leber, männliche Reproduktionsorgane, pränatale Exposition von Mäusen u. Ratten in anästhetisch wirksamen Konzentrationen: postnatale Effekte auf Verhalten (Maus: niedrigste inhalative Effektkonzentration 4000 ml/m^3^; Ratte: 13 000 ml/m^3^); höhere Empfindlichkeit für neurotoxische Effekte von Nagern u. Makaken im neonatalen u. juvenilen Alter als im Erwachsenenalter; 7 d alte Mäuse u. Ratten nach einmaliger Exposition gegenüber anästhetisch wirksamer Konzentration (7500 ml/m^3^): erhöhte Neuroapoptose u. Beeinträchtigungen von Lernen u. Gedächtnis im Erwachsenenalter; mechanistische In-vitro-Studien: Hinweise auf sensitive Reaktion des sich entwickelnden Gehirns mit Neurodegenerationen	niedrigste inhalative Effektkonzentration (Maus) 4000 ml/m^3^: 1000-facher Abstand zum MAK-Wert von 2 ml/m^3^ (Berücksichtigung des erhöhten Atemvolumens, 1:2)	Hartwig und MAK Commission2022 a

d: Tag; i.p.: intraperitoneal; u.: und; V: Vanadium; Vgl.: Vergleich

Im Folgenden werden zwei Stoffbeispiele näher dargestellt.

Die Bewertung von **Vanadium** ist vor der Einführung der Gruppe B (Verdacht) vorgenommen worden:

Zu Vanadium reichen die Daten zur pränatalen Entwicklungstoxizität aus, um eine Zuordnung zu Gruppe C vornehmen zu können. In-vitro- und In-vivo-Versuche zeigen jedoch eine höhere Empfindlichkeit von Oligodendrozytenvorläuferzellen im Vergleich zu reifen Oligodendrozyten sowie Störungen der Myelinisierung und motorische Beeinträchtigungen bei neugeborenen/juvenilen Ratten. Da nur eine Dosis verabreicht wurde, gibt es keinen NOAEL für die entwicklungsneurotoxischen Effekte. Für die effektive In-vivo-Dosis von 1,25 mg Vanadium/kg KG und Tag nach intraperitonealer Gabe, dem Worst Case, lässt sich eine Luftkonzentration von 2,2 mg Vanadium/m^3^ abschätzen (intraperitoneale und inhalative Aufnahme je 100 %), was einem 440-fachen Abstand zum MAK-Wert entspricht. Somit liegt zunächst ein Verdacht auf eine entwicklungsneurotoxische Wirkung vor, aber aufgrund der fehlenden NOAEC für diesen Effekt kann keine Risikobewertung und folglich keine Zuordnung zur Schwangerschaftsgruppe A, B oder C vorgenommen werden. Daher ist der Stoff der Schwangerschaftsgruppe D zugeordnet worden (Hartwig und MAK Commission^2023^).

Die Prüfung einer möglichen Zuordnung zu Gruppe B (Verdacht) hat für Vanadium Folgendes erbracht:

Bei Vanadium wurde die MAK-Wert-Ableitung nicht von einem neurotoxischen Effekt, sondern ausgehend von der Lungentoxizität als kritischem Effekt abgeleitet. Der MAK-Wert ist mit 0,005 mg Vanadium/m^3^ E als vergleichsweise niedrig anzusehen. Neurotoxische Effekte wurden bei Beschäftigten, die länger als ein Jahr in der Herstellung vanadiumhaltiger Produkte tätig waren, bei einer mittleren Konzentration von 0,216 mg Vanadium/m^3^ beobachtet. In tierexperimentellen Studien kam es nach inhalativer und nach intraperitonealer Exposition zu neurotoxischen Effekten und histopathologischen Veränderungen bei 0,78 mg Vanadium/m^3^ (adulte Mäuse) bzw. 1,25 mg Vanadium/kg KG und Tag (neugeborene, juvenile Ratten, siehe oben) (Hartwig und MAK Commission^2023^). Auch wenn keine NOAEL bekannt sind, ist ein relativer großer Abstand zwischen Effektkonzentration bzw. Effektdosis und MAK-Wert (156- bzw. 251-fach) gegeben, sodass eine mögliche entwicklungsneurotoxische Wirkung als unwahrscheinlich anzusehen ist.

Auch die Bewertung von **Isofluran** ist vor der Einführung der Gruppe B (Verdacht) vorgenommen worden:

Für Isofluran ist auf Basis der Daten zur pränatalen Entwicklungstoxizität eine Zuordnung zu Gruppe C möglich. Jedoch verursacht die pränatale Exposition postnatal Effekte im Verhalten bei Mäusen (niedrigste Effektkonzentration 4000 ml/m^3^) und Ratten (niedrigste Effektkonzentration 13 000 ml/m^3^), was nur bei anästhetisch wirksamen Konzentrationen getestet worden ist. Zudem reagieren Nager und Makaken im neonatalen und juvenilen Alter empfindlicher auf die neurotoxischen Effekte von Isofluran als im Erwachsenenalter. So kommt es bei sieben Tage alten Mäusen und Ratten nach einmaliger Exposition gegen eine anästhetisch wirksame Konzentration von 7500 ml/m^3^ zu erhöhter Neuroapoptose und zu Beeinträchtigungen von Lernen und Gedächtnis im Erwachsenenalter. Es liegen keine Studien an neonatalen oder juvenilen Tieren mit nicht-anästhetischen Konzentrationen vor, aus denen sich eine NOAEC für entwicklungsneurotoxische Effekte von Isofluran ableiten lassen würde. Auch mechanistische In-vitro-Studien geben Hinweise, dass das sich entwickelnde Gehirn sensitiv auf Isofluran mit Neurodegenerationen reagiert. Somit ergibt sich der Verdacht auf eine entwicklungsneurotoxische Wirkung. Allerdings fehlen Untersuchungen bei nicht-anästhetischen Konzentrationen. Aufgrund der fehlenden NOAEC für diesen Effekt kann keine Risikobewertung (d. h. keine Zuordnung zu einer Risiko-basierten Schwangerschaftsgruppe) vorgenommen werden und der Stoff ist der Schwangerschaftsgruppe D zugeordnet worden (Hartwig und MAK Commission2022 a).

Die Prüfung einer möglichen Zuordnung zu Gruppe B (Verdacht) hat für Isofluran Folgendes erbracht:

Auch bei Isofluran war der kritische Endpunkt für die MAK-Wert-Ableitung nicht die Neurotoxizität, sondern Effekte auf die Leber und männliche Reproduktionsorgane bei Ratten. Die niedrigste inhalative Effektkonzentration für entwicklungsneurotoxische Wirkungen (Maus) beträgt 4000 ml/m^3^ und liegt im Bereich beginnender Anästhesie der Muttertiere (Hartwig und MAK Commission2022 a). Mit einem 1000-fachen Abstand zum MAK-Wert von 2 ml/m^3^ (Berücksichtigung des erhöhten Atemvolumens, 1:2) und der Ableitung des MAK-Wertes ausgehend von einem nicht-neurotoxischen Endpunkt ist ein „Puffer“ gegeben, der das entwicklungsneurotoxische Potenzial deutlich vermindert.

**Fazit**: Für alle Stoffe der Tabelle 1 ergibt sich zunächst ein Verdacht auf eine entwicklungsneurotoxische Wirkung. Bei Vanadium und Isofluran wird der Verdacht jedoch dadurch relativiert, dass der MAK-Wert nicht auf der Neurotoxizität basiert und die Neurotoxizität erst bei höheren Konzentrationen beginnt. Für die anderen Stoffe hat die Überprüfung nicht genügend Hinweise erbracht, um den anfänglichen Verdacht auf eine entwicklungsneurotoxische Wirkung aufrecht zu erhalten.

## Schlussfolgerung zur Gruppe B (Verdacht)

Für einen Stoff werden die Argumente für und gegen einen Verdacht auf eine entwicklungs(neuro)toxische Wirkung zusammengestellt. Diese Argumente werden durch ein Expert-Judgement geprüft. Dabei ist darauf zu achten, ob die Annahme eines realistischen Gefahrenpotenzials bzw. Verdachts auf eine fruchtschädigende Wirkung für den Menschen ausreichend plausibel ist.

## ANHANG

### Text aus der MAK- und BAT-Werte-Liste 2025: VIII. MAK-Werte und Schwangerschaft

Die Einhaltung von MAK- und BAT-Werten gewährleistet nicht in jedem Falle den sicheren Schutz des ungeborenen Kindes, da zahlreiche Arbeitsstoffe nicht oder nur teilweise auf fruchtschädigende Wirkungen untersucht worden sind.

#### Definition

Der Begriff „fruchtschädigend“ bzw. „entwicklungstoxisch“ wird von der Kommission im weitesten Sinne verstanden, und zwar im Sinne jeder Stoffeinwirkung, die eine gegenüber der physiologischen Norm veränderte Entwicklung des Organismus hervorruft, die prä- oder postnatal zum Tod oder zu einer permanenten morphologischen oder funktionellen Schädigung des Ungeborenen führt.

#### Erfahrungen beim Menschen

Epidemiologische Studien, die Hinweise auf fruchtschädigende Wirkungen von Stoffen beim Menschen geben, sind für die Bewertung von besonderer Bedeutung. Limitierungen solcher Studien wie methodische Schwächen, geringe statistische Aussagekraft, Mischexposition, persönliche Einflussfaktoren und Lebensstil erschweren eine eindeutige Aussage über stoffspezifische Wirkungen und Effektschwellen.

#### Tierexperimentelle Untersuchungen

Die Beurteilung der entwicklungstoxischen Eigenschaften von Substanzen erfolgt überwiegend auf Grundlage von tierexperimentellen Studien. Von maßgeblicher Bedeutung sind hierbei Studien, die nach international anerkannten Prüfrichtlinien, wie den OECD- oder vergleichbaren Prüfrichtlinien (z. B. EU, Japan), durchgeführt wurden. Zur Ermittlung der pränatalen Toxizität ist vor allem die OECD‐Prüfrichtlinie 414 relevant. Die Prüfung der peri‐ und postnatalen Toxizität, in eingeschränktem Maß auch der pränatalen Toxizität, erfolgt vor allem in Ein-Generationenstudien nach OECD‐Prüfrichtlinie 415 (bis 27.12.2019 gültig), in erweiterten Ein-Generationenstudien nach OECD‐Prüfrichtlinie 443, in Zwei-Generationenstudien nach OECD‐Prüfrichtlinie 416 oder in Screening‐Tests nach den OECD-Prüfrichtlinien 421 und 422. Liegen Studien vor, die nicht nach diesen Richtlinien durchgeführt wurden, ist deren Aussagekraft im Einzelnen zu bewerten. Die wichtigsten Kriterien hierfür sind eine ausreichend große Tierzahl, die Verwendung verschiedener Dosisgruppen mit der Ableitung eines NOAEL (No Observed Adverse Effect Level), ausreichende Untersuchungstiefe (äußere, skelettale und viszerale Untersuchungen der Feten bei den Entwicklungstoxizitätsstudien) und eine ausreichende Dokumentation der Befunde.

Zur Beurteilung der fruchtschädigenden Wirkungen von Stoffen am Arbeitsplatz sind Inhalationsstudien von besonderer Bedeutung. Jedoch können auch Studien mit oraler oder dermaler Verabreichung berücksichtigt werden, wenn die vorhandenen Daten nicht gegen eine Übertragung auf die inhalative Situation sprechen (z. B. bei einem ausgeprägten First-Pass-Effekt). Studien, die mit Applikationswegen durchgeführt wurden, die für den Menschen nicht relevant sind (z. B. intraperitoneal) werden in der Regel für die Bewertung nicht herangezogen.

Bei Studien mit oraler Verabreichung sind meist höhere Dosierungen möglich als mit inhalativer oder dermaler Verabreichung. Damit werden auch Effekte erfasst, die nur im hohen Dosisbereich auftreten. In den genannten Prüfrichtlinien gelten daher 1000 mg/kg KG als maximal zu testende Dosierung („limit dose“). Solche Hochdosiseffekte sind für die Beurteilung von fruchtschädigenden Wirkungen bei Konzentrationen im Bereich des MAK‐Wertes meist ohne Relevanz. Von geringer Relevanz für die Situation am Arbeitsplatz sind Fruchtschädigungen, die in Gegenwart ausgeprägter maternaler Toxizität zu beobachten sind, da diese durch Einhaltung des MAK‐Wertes verhindert werden. Von besonderer Relevanz sind Befunde in Dosierungen bzw. Konzentrationen, bei denen keine oder nur geringfügige maternale Toxizität zu beobachten ist.

Als bevorzugte Versuchstierspezies werden in der oben genannten Prüfrichtlinie zur pränatalen Entwicklungstoxizität (OECD 414) üblicherweise weibliche Ratten und Kaninchen empfohlen. Dagegen werden die Generationenstudien (z. B. OECD 415, 416 und 443) einschließlich der Screening‐Tests (z. B. OECD 421 und 422) normalerweise nur mit Ratten beiderlei Geschlechts durchgeführt. Zur Berücksichtigung des unterschiedlichen Reifegrades von Organen bei der Geburt des Nagers im Vergleich zum Menschen (z. B. Gehirn und Nieren) wird zur Bewertung der Entwicklungstoxizität bei der Untersuchung von Nagern gegebenenfalls die Exposition bis in den Postnatalbereich hinein betrachtet.

Um Unsicherheiten in der Bewertung der Tierversuche zu berücksichtigen, ist ein ausreichender Abstand zwischen dem NOAEL für entwicklungstoxische Effekte im Tierexperiment und der resultierenden Belastung bei Einhaltung des MAK‐ bzw. BAT‐Wertes erforderlich. Die erforderliche Größe des Abstandes hängt von einer Anzahl sehr unterschiedlicher Faktoren ab:

- Vergleichende toxikokinetische Daten bei Mensch und Tier.
- Kenntnis des toxikokinetischen Profils eines Stoffes bei Muttertier und Embryonen bzw. Feten, um Unterschiede in der Belastung zwischen maternalen und fetalen Organen/Geweben zu beurteilen.
- Liegen solche Daten nicht vor, spielt die Beurteilung spezifischer Stoffeigenschaften, wie Molekülgröße, Lipidlöslichkeit und Proteinbindung, eine wesentliche Rolle, weil diese für den transplazentaren Übergang des Stoffes vom Muttertier maßgeblich sind und die innere Belastung der Embryonen bzw. Feten bestimmen.
- Art und Schweregrad der beobachteten Befunde sind wichtige Faktoren. So sind gravierende Effekte, wie das vermehrte Vorkommen spezifischer Fehlbildungen in Dosierungen ohne gleichzeitige maternale Toxizität stärker zu berücksichtigen als eher transiente unspezifische bzw. weniger schwerwiegende fetotoxische Effekte, wie geringfügig verringertes fetales Körpergewicht oder verzögerte Skelettreifung. Die Festlegung des erforderlichen Abstandes ist somit ein stoffspezifischer Prozess, der zu unterschiedlich begründeten Ergebnissen führt.

#### Berücksichtigung der Entwicklungsneurotoxizität

Seit 2016 wird für Substanzen, deren MAK- oder BAT-Wert von einem neurotoxischen Effekt abgeleitet worden ist, eine Aussage über entwicklungsneurotoxische Effekte getroffen. Eine Betrachtung der Entwicklungsneurotoxizität wird auch in folgendem Falle vorgenommen: Wenn der kritische Effekt für die MAK- oder BAT-Wert-Ableitung nicht die Neurotoxizität ist, eine Substanz jedoch neurotoxisch wirkt und sich neonatale bzw. juvenile Tiere im Vergleich zu adulten Tieren als empfindlicher für die substanzinduzierten neurotoxischen Effekte erwiesen haben. Am geeignetsten für die Bewertung der Entwicklungsneurotoxizität sind Studien mit pränataler Exposition und Untersuchung neurotoxischer Endpunkte bei heranwachsenden bzw. adulten Nachkommen unter Beachtung des bei adulten Tieren aufgetretenen Effekts, wie zum Beispiel Studien nach Prüfrichtlinien zur Entwicklungsneurotoxizität (OECD-Prüfrichtlinie 426) oder erweiterte Ein-Generationenstudien (OECD-Prüfrichtlinie 443) sowie weitere geeignete Studien zur Entwicklungsneurotoxizität. Zudem werden entsprechende Informationen zu Toxikokinetik und Wirkungsmechanismus herangezogen. Die Bewertung einer möglichen entwicklungsneurotoxischen Wirkung wird bei der Zuordnung zur Schwangerschaftsgruppe mitberücksichtigt.

#### Kennzeichnung als Verdacht auf fruchtschädigende Wirkung (Gruppe B (Verdacht))

Für Substanzen, die aufgrund fehlender Daten oder unvollständiger Untersuchung zur Entwicklungs(neuro)toxizität nicht eindeutig der Schwangerschaftsgruppe A, B oder C zugeordnet werden können, ist bisher eine Zuordnung zur Schwangerschaftsgruppe D erfolgt. Bei Substanzen, für die kein MAK- oder BAT-Wert (Zuordnung zum Abschnitt II b) aufgestellt werden kann bzw. die in Kanzerogenitäts-Kategorie 1, 2, 3 (ohne MAK-Wert) eingestuft worden sind, erfolgte bisher keine Bewertung der fruchtschädigenden Wirkung. Für diese Substanzen ist zur Kennzeichnung einer möglichen fruchtschädigenden Wirkung 2024 eine Gruppe B (Verdacht) eingeführt worden. Damit wird verdeutlicht, dass es Gründe zur Annahme eines Gefahrenpotenzials/Verdachts gibt, aber die vorliegenden Daten zur Risikoabschätzung zum MAK- bzw. BAT-Wert fehlen bzw. nicht ausreichen.

Ein Gefahrenpotenzial kann sich aus mechanistischen Hinweisen aus In-vivo-Untersuchungen, meist nach OECD-Prüfrichtlinie 421, 422, 426 oder 443 und evtl. aus In-vitro-Untersuchungen ergeben. Bei der Beurteilung sollte geprüft werden, ob die Annahme eines realistischen Gefahrenpotenzials bzw. Verdachts für den Menschen ausreichend plausibel ist.

#### Schwangerschaftsgruppen

Auf Basis der genannten Voraussetzungen überprüft die Kommission alle gesundheitsschädlichen Arbeitsstoffe mit MAK‐ oder BAT‐Wert daraufhin, ob eine fruchtschädigende Wirkung bei Einhaltung des MAK‐ oder BAT‐Wertes nicht anzunehmen ist (Gruppe C), ob eine solche nach den vorliegenden Informationen nicht auszuschließen ist (Gruppe B) oder sicher nachgewiesen ist (Gruppe A). Wenn die vorliegenden Daten nicht für eine belastbare Risikoabschätzung zum MAK- bzw. BAT-Wert ausreichen, wird seit 2024 für jede Substanz geprüft, ob ein Verdacht auf eine fruchtschädigende Wirkung besteht (Gruppe B (Verdacht)). Für eine Anzahl an Arbeitsstoffen ist es jedoch vorerst nicht möglich, eine Aussage zur fruchtschädigenden Wirkung zu treffen (Gruppe D). Dies ist in der jeweiligen Begründung ausführlich dargestellt.

Folgende Schwangerschaftsgruppen werden daher definiert:

**Gruppe A: **Eine fruchtschädigende Wirkung ist beim Menschen sicher nachgewiesen und auch bei Einhaltung des MAK‐ und BAT‐Wertes zu erwarten.

**Gruppe B: **Eine fruchtschädigende Wirkung ist nach den vorliegenden Informationen bei Exposition in Höhe des MAK‐ und BAT‐Wertes nicht auszuschließen. In der jeweiligen Begründung ist, sofern die Bewertung der Datenlage durch die Kommission es ermöglicht, ein Hinweis gegeben, welche Konzentration der Zuordnung zur Schwangerschaftsgruppe C entsprechen würde. Die Stoffe mit einem Hinweis werden in der MAK- und BAT‐Werte‐Liste mit der Fußnote *„Hinweis auf Voraussetzung für Gruppe C siehe Begründung“ *versehen.

**Gruppe B (Verdacht): **Es ergibt sich aus den vorliegenden Daten ein Verdacht auf eine fruchtschädigende Wirkung, aber die vorliegenden Daten reichen nicht für eine belastbare Risikoabschätzung zum MAK- oder BAT-Wert aus.

**Gruppe C: **Eine fruchtschädigende Wirkung ist bei Einhaltung des MAK‐ und BAT-Wertes nicht anzunehmen.

**Gruppe D: **Für die Beurteilung der fruchtschädigenden Wirkung, ggf. inklusive der entwicklungsneurotoxischen Wirkung, liegen entweder keine Daten vor oder die vorliegenden Daten reichen für eine Einstufung in eine der Gruppen A, B oder C bzw. Gruppe B (Verdacht) nicht aus.

Arbeitsstoffe ohne MAK‐ oder BAT‐Wert (krebserzeugende Stoffe oder Stoffe des Abschnittes II b) erhalten ein „−“ oder gegebenenfalls eine Zuordnung zu Gruppe B (Verdacht).

**Tab. 2:** Auswertung der Stoffe mit Zuordnung zu Schwangerschaftsgruppe D (Stand: 01.07.2024)

**Eintrag**	**Anzahl der Einträge**	**Prozentsatz der Einträge **
Einträge in Gruppe D	125	100 %
Grund für Eingruppierung in D:		
Keine Daten zur Entwicklungstoxizität	81	65 %
Keine validen Daten zur Entwicklungstoxizität	15	12 %
Nur Studie nach OECD 422, keine Untersuchung zur Teratogenität	14	11 %
Nur Studie nach OECD 421, keine Untersuchung zur Teratogenität	3	2 %
Keine oder keine validen Daten zur Entwicklungsneurotoxizität	12	10 %

## References

[ref_359SALZT] AGS (Ausschuss für Gefahrstoffe) (2025) Technische Regeln für Gefahrstoffe (TRGS 900). Arbeitsplatzgrenzwerte. Dortmund: BAuA. https://www.baua.de/DE/Angebote/Regelwerk/TRGS/pdf/TRGS-900.pdf?__blob=publicationFile&v=11, abgerufen am 06 Aug 2025

[ref_SYQW5LTT] Greim H, Hrsg (1998) Xylidin (Isomeren). In: Gesundheitsschädliche Arbeitsstoffe, Toxikologisch-arbeitsmedizinische Begründung von MAK-Werten. 27. Lieferung. Weinheim: Wiley-VCH. Auch erhältlich unter https://doi.org/10.1002/3527600418.mb130073ismd0027

[ref_QUDHYAKJ] Hartwig A, Hrsg (2009) Naphtha (Erdöl), mit Wasserstoff behandelte schwere. In: Gesundheitsschädliche Arbeitsstoffe, Toxikologisch-arbeitsmedizinische Begründung von MAK-Werten. 48. Lieferung. Weinheim: Wiley-VCH. Auch erhältlich unter https://doi.org/10.1002/3527600418.mb6474248yold0048

[ref_C7YD6HYW] Hartwig A, Hrsg (2015) Dichlormethan. In: Gesundheitsschädliche Arbeitsstoffe, Toxikologisch-arbeitsmedizinische Begründung von MAK-Werten. 59. Lieferung. Weinheim: Wiley-VCH. Auch erhältlich unter https://doi.org/10.1002/3527600418.mb7509d0059

[ref_EVZZ9GHB] Hartwig A, MAK Commission (2017) Trimethylpentan (alle Isomere). MAK Value Documentation in German language. MAK Collect Occup Health Saf 2(2): 1015-1031. https://doi.org/10.1002/3527600418.mb2922248isd0063

[ref_ZG69BHAE] Hartwig A, MAK Commission (2018) 1,2,4-Triethylbenzol. MAK-Begründung. MAK Collect Occup Health Saf 3(2): 831–838. https://doi.org/10.34865/mb87744d0065_w]

[ref_IX4MPLHS] Hartwig A, MAK Commission (2020 a) Trikresylphosphat, Summe aller o-Isomere. MAK-Begründung. MAK Collect Occup Health Saf 5(3): Doc050. https://doi.org/10.34865/mb7830d5_3or

[ref_HWYHNEI5] Hartwig A, MAK Commission (2020 b) Xylidin, Isomere (2,3-Xylidin, 2,5-Xylidin, 3,4-Xylidin, 3,5-Xylidin). MAK-Begründung, Nachtrag. MAK Collect Occup Health Saf 5(2): Doc033. https://doi.org/10.34865/mb8759ismd5_2ad PMC13510802 42657357

[ref_QQ6TTQV7] Hartwig A, MAK Commission (2021 a) Chlormethan. MAK-Begründung, Nachtrag. MAK Collect Occup Health Saf 6(3): Doc049. https://doi.org/10.34865/mb7487d6_3ad

[ref_8ZP4YAEY] Hartwig A, MAK Commission (2021 b) Xylol (alle Isomere). MAK-Begründung, Nachtrag. MAK Collect Occup Health Saf 6(1): Doc004. https://doi.org/10.34865/mb133020d6_1ad PMC13502067 42639483

[ref_4W8QIK2B] Hartwig A, MAK Commission (2022 a) Isofluran. MAK-Begründung, Nachtrag. MAK Collect Occup Health Saf 7(3): Doc044. https://doi.org/10.34865/mb2667546d7_3ad

[ref_V2A8MJIY] Hartwig A, MAK Commission (2022 b) Triphenylphosphin. MAK-Begründung, Nachtrag. MAK Collect Occup Health Saf 7(3): Doc047. https://doi.org/10.34865/mb60335d7_3ad

[ref_TTHNS6J3] Hartwig A, MAK Commission (2023) Vanadium und seine anorganischen Verbindungen. MAK-Begründung, Nachtrag. MAK Collect Occup Health Saf 8(4): Doc076. https://doi.org/10.34865/mb744062d8_4ad

[ref_LNYKF8ZS] Hartwig A, MAK Commission (2025 a) 1,1-Dichlorethen. MAK-Begründung, Nachtrag. MAK Collect Occup Health Saf 10(2): Doc022. https://doi.org/10.34865/mb7535d10_2ad 41586717 PMC12443063

[ref_CPHBA6ER] Hartwig A, MAK Commission (2025 b) 1,3-Bis(hydroxymethyl)harnstoff. MAK-Begründung, Nachtrag. MAK Collect Occup Health Saf 10(1): Doc003. https://doi.org/10.34865/mb14095kskd10_1ad 41586725 PMC12823113

[ref_HPP3KKT7] Hartwig A, MAK Commission (2025 c) 4-(2-Nitrobutyl)morpholin und 4,4′-(2-Ethyl-2-nitro-1,3-propandiyl)bismorpholin. MAK-Begründung, Nachtrag. MAK Collect Occup Health Saf 10(2): Doc026. https://doi.org/10.34865/mb222444kmxd10_2ad 41586710 PMC12443061

[ref_PRP3V4X9] Hartwig A, MAK Commission (2025 d) Allylpropyldisulfid. MAK-Begründung, Nachtrag. MAK Collect Occup Health Saf 10(1): Doc001. https://doi.org/10.34865/mb217959d10_1ad 41586721 PMC12823114

[ref_Y8TWZ9DE] Hartwig A, MAK Commission (2025 e) Anilin. MAK-Begründung, Nachtrag. MAK Collect Occup Health Saf 10(1): Doc002. https://doi.org/10.34865/mb6253d10_1ad 41586722 PMC12823116

[ref_DGSB589P] Hartwig A, MAK Commission (2025 f) Bis(morpholino)methan. MAK-Begründung, Nachtrag. MAK Collect Occup Health Saf 10(1): Doc004. https://doi.org/10.34865/mb562590kskd10_1ad 41586733 PMC12823118

[ref_L4ZVHHEK] Hartwig A, MAK Commission (2025 g) Methenamin-3-chlorallylchlorid. MAK-Begründung, Nachtrag. MAK Collect Occup Health Saf 10(1): Doc006. https://doi.org/10.34865/mb408031kskd10_1ad 41586732 PMC12820731

[ref_SXUR6TCK] Hartwig A, MAK Commission (2025 h) N,N´,N´´-Triethylhexahydro-1,3,5-triazin. MAK-Begründung, Nachtrag. MAK Collect Occup Health Saf 10(2): Doc027. https://doi.org/10.34865/mb777927kskd10_2ad 41586700 PMC12443066

[ref_73DT64GE] Hartwig A, MAK Commission (2025 i) Naphtha (Erdöl), mit Wasserstoff behandelte schwere. MAK-Begründung, Nachtrag. MAK Collect Occup Health Saf 10(2): Doc025. https://doi.org/10.34865/mb6474248yold10_2ad 41586704 PMC12443067

[ref_VUYNVHFM] Hartwig A, MAK Commission (2025 j) N-Methylolchloracetamid. MAK-Begründung, Nachtrag. MAK Collect Occup Health Saf 10(2): Doc024. https://doi.org/10.34865/mb283219kskd10_2ad 41586712 PMC12443134

[ref_5CN87PCK] Hartwig A, MAK Commission (2026 a) 1,3-Dimethylol-5,5′-dimethylhydantoin. MAK-Begründung, Nachtrag. MAK Collect Occup Health Saf 11(1): Doc005. https://doi.org/10.34865/mb644058kskd11_1ad 42639475 PMC13502082

[ref_MWE6YZMW] Hartwig A, MAK Commission (2026 b) Benzophenon-3. MAK-Begründung. MAK Collect Occup Health Saf 11(2): Doc020. https://doi.org/10.34865/mb13157d11_2or PMC13510973 42657204

[ref_YBJV7BY5] Hartwig A, MAK Commission (2026 c) Benzylalkoholmono(poly)hemiformal. MAK-Begründung, Nachtrag. MAK Collect Occup Health Saf 11(1): Doc002. https://doi.org/10.34865/mb1454860kskd11_1ad 42639090 PMC13502063

[ref_HJAVAU2H] Hartwig A, MAK Commission (2026 d) N,N-Bis(2-ethylhexyl)-((1,2,4-triazol-1-yl)methyl)amin. MAK-Begründung, Nachtrag. MAK Collect Occup Health Saf 11(1): Doc003. https://doi.org/10.34865/mb9127304ksd11_1ad 42639481 PMC13502076

[ref_QYZIGRWT] Hartwig A, MAK Commission (2026 e) Xylidin, Isomere (2,3-Xylidin, 2,5-Xylidin, 3,4-Xylidin, 3,5-Xylidin). MAK-Begründung, Nachtrag. MAK Collect Occup Health Saf 11(2): Doc025. https://doi.org/10.34865/mb8759ismd11_2ad PMC13510802 42657357

[ref_QFK736PC] Leng G, Bolt HM (2008) Methämoglobin-Bildner. In: Biologische Arbeitsstoff-Toleranz-Werte (BAT-Werte), Expositionsäquivalente für krebserzeugende Arbeitsstoffe (EKA) und Biologische Leitwerte (BLW). 15. Lieferung. Weinheim: Wiley-VCH. Auch erhältlich unter https://doi.org/10.1002/3527600418.bb6253d0015

[ref_5QMS9UBZ] Semple BD, Blomgren K, Gimlin K, Ferriero DM, Noble-Haeusslein LJ (2013) Brain development in rodents and humans: identifying benchmarks of maturation and vulnerability to injury across species. Prog Neurobiol 106-107: 1-16. https://doi.org/10.1016/j.pneurobio.2013.04.001 23583307 PMC3737272

